# Identification of the CesA Subfamily and Functional Analysis of GhMCesA35 in *Gossypium hirsutum* L.

**DOI:** 10.3390/genes13020292

**Published:** 2022-02-01

**Authors:** Ruolin Zhao, Hailiang Cheng, Qiaolian Wang, Limin Lv, Youping Zhang, Guoli Song, Dongyun Zuo

**Affiliations:** 1 Institute of Cotton Research of Chinese Academy of Agricultural Sciences, Anyang 455000, China; 18853867163@163.com (R.Z.); pser2010@163.com (H.C.); wangql1232021@163.com (Q.W.); llm0372@126.com (L.L.); zyp547550790@163.com (Y.Z.); sglzms@163.com (G.S.); 2 Zhengzhou Research Base, State Key Laboratory of Cotton Biology, Zhengzhou University, Zhengzhou 450001, China

**Keywords:** cotton, CesA, cellulose, gene family, expression pattern

## Abstract

The cellulose synthase genes control the biosynthesis of cellulose in plants. Nonetheless, the gene family members of CesA have not been identified in the newly assembled genome of *Gossypium* *hirsutum* (AD1, HEBAU_NDM8). We identified 38 CesA genes in *G. hirsutum* (NDM8) and found that the protein sequence of GhMCesA35 is 100% identical to CelA1 in a previous study. It is already known that CelA1 is involved in cellulose biosynthesis in vitro. However, the function of this gene in vivo has not been validated. In this study, we verified the function of GhMCesA35 in vivo based on overexpressed *Arabidopsis thaliana*. In addition, we found that it interacted with GhCesA7 through the yeast two-hybrid assay. This study provides new insights for studying the biological functions of CesA genes in *G. hirsutum*, thereby improving cotton fiber quality and yield.

## 1. Introduction

Cotton is a significant source of natural fiber in the world [1]. *G. hirsutum,* the upland cotton, accounts for more than 95% of cotton fiber production [2]. Therefore, research about upland cotton’s fiber development is essential for cotton molecular breeding.

Cotton fiber is a unique epidermal hair cell of cotton, which develops from a single cell and has four developmental stages: initiation, elongation, secondary wall thickening, and maturity [3]. The process of secondary wall thickening is accompanied by cell wall expansion in which synthesis and deposition of cellulose play key roles [4]. At this stage, the rate of cellulose synthesis is estimated to increase nearly 100 times [5]. Given this, the cellulose in cotton had been investigated, and insights were provided into fiber development.

Cellulose plays a central role in determining the mechanical properties of plant cell walls. It is important for the cell wall’s strength and hardness [6,7]. Therefore, a large amount of cellulose is essential for cells to maintain the various functions of the cell wall, including regulating cell expansion, promoting cell adhesion, forming resistance to biotic and abiotic stresses, and determining the physical properties of plant tissues [8,9,10,11]. As for cellulose in cotton, numerous celluloses accumulate in the mutual fibers, influencing the final yield and quality of cotton fibers [12,13]. Cellulose, as a polysaccharide, consists of β-1,4-linked glucose units and is generated by the cellulose synthase complex (CSC) on the cell membrane. In a previous study, a freeze-etching section of the plasma membrane showed that CSC is a rosette structure composed of six subunits [14]. Each subunit contained six monomers, and each monomer synthesized a glucan chain. In total, each CSC can form 36 cellulose microfibrils, which are integrated into a single cellulose molecule [15]. Apart from CSC with 6 subunits, CSC with 3 subunits was also reported, and this kind of CSC can produce 18 cellulose microfibrils [16,17,18]. The cellulose synthase superfamily, belonging to the glycosyltransferase (GT) family 2, mainly includes the cellulose synthase (CesA) gene family and cellulose synthase-like protein (Csl) gene family [19]. CSL can be divided into 10 families (CSLA, B, C, D, E, F, G, H, J, and K) based on the early phylogenetic studies of CesA homologues in model plants [20]. In *Arabidopsis thaliana,* 10 CesA genes have been identified [21]. AtCesA1 and AtCesA3 are necessary for cell growth and control cellulose synthesis in the primary cell wall (PCW). On the other hand, AtCesA4, AtCesA7, and AtCesA8 participate in secondary cell wall synthesis [22,23]. Null mutations in AtCesA1 and AtCesA3 are lethal [24]. For genes involved in secondary cell wall synthesis, AtCesA8 is coexpressed with AtCesA4 and AtCesA7 to form a cellulose synthase complex [25,26]. In cotton, CelA1 and CelA2 are involved in secondary cell wall biosynthesis and bind UDP-glucose in vitro [27]. In addition, GhCesA4 was isolated from cotton fiber and analyzed by Northern blot. The results showed that GhCesA4 was specifically expressed in fiber tissues [28].

Although, some CesA genes have been identified in cotton. One obvious challenge is obtaining definitive proof that these genes are truly functional in cellulose synthesis in vivo. Moreover, the protein sequences of GhMCesA35 and CelA1 are 100% identical. Therefore, we selected GhMCesA35 for functional analysis, which is highly expressed during the secondary wall thickening stage by transcriptome data combined with qRT-PCR. The cloning and identification of GhMCesA35 lays a foundation for improving cotton fiber quality and provides insights on the mechanism of cotton cellulose synthesis.

## 2. Materials and Methods

### 2.1. Plant Materials

The cotton plant used in this experiment was upland cotton TM-1, grown in the experimental field of Cotton Research Institute of Chinese Academy of Agricultural Sciences at Anyang, Henan, China. The roots, stems, and leaves of Hai7124 were collected at the cotton seedling stage. Additional ovules and fiber samples of TM-1 were collected at 0, 5, 10, 20, 25, and 30 days post-anthesis (DPA). However, the fiber samples were collected at 0, 10, 20, 25, and 30 days post-anthesis (DPA). Colombian wild-type *A. thaliana* and *N. tabacum* (benthamiana) seeds were surface sterilized with 1% sodium hypochlorite followed by 75% ethanol. The sterilized seeds were placed on 0.8% MS agar medium and stratified at 4 °C for 3 days in the dark, and transferred to growth condition with a photoperiod of 16-h light/8-h dark.

### 2.2. Sequence Retrieval and Identification of CesA Genes in Gossypium

The CesA gene family protein sequences of *Arabidopsis* were obtained from *TAIR* [29]. We downloaded the genome sequence files and genetic feature format files of *G. hirsutum* acc. NDM8 (AD1, HEBAU_NDM8) from CottonGEN [30]. In addition, to ensure the consistency of the gene ID, we used TBtools to combine genetic features format (gff3) files and genome sequence files to generate protein sequence files [31]. First, to identify cotton cellulose synthase family members, the profile hidden Markov model (PF03552.11) of the HMMER was applied to search the protein sequence data [32]. Then, the 10 protein sequences of the candidate CesA gene family members in *A. thaliana* were used as the query sequence. BLASTP (E-value = 1 × 10^−5^ arches were performed in the above genome databases TBtools [31]. The default parameter settings were used. Then, we removed the redundancy of the sequences obtained in the above two steps, and candidate cellulose synthase protein sequences were submitted to CottonFGD (https://cottonfgd.org/, accessed on 25 January 2022) [33]. Finally, 38 CesA genes were selected and submitted to Pfam search (Batch sequence search) for domain confirmation (https://pfam.xfam.org/search#tabview=tab1, accessed on 25 January 2022) according to the gene name [34], e-value = 1 × 10^−5^. The gene IDs and numbers involved in each step are provided in the Supplementary Materials Table S4. Using the ExPASy online tools (https://web.expasy.org/compute_pi/, accessed on 25 January 2022), we computed the theoretical pI (isoelectric point) and Mw (molecular weight) [35]. Using the CottonFGD (https://cottonfgd.org/, accessed on 25 January 2022), we obtained the information regarding the transcript ID, gene name, description, chromosome, start, end strand, gene length and GO list, etc. of the CesA genes [33].

### 2.3. Sequence Analysis and Phylogenetic Tree Construction

Multiple sequence alignments of all cellulose synthase protein sequences in *G. hirsutum* and *Arabidopsis thaliana* were performed using Muscle Wrapper in TBtools software (version 1.098691) with the default settings [31]. Then, using trimAL Wrapper in TBtools software, the multiple sequence alignment results were clipped. Finally, the maximum likelihood (ML) method was employed to construct phylogenetic trees using the same software. The number of bootstraps was 1000. The phylogenetic tree was constructed on the Newick file generated by MEGA through the online website EvolView [36].

### 2.4. Chromosomal Location, Genetic Structure, and Motif Analysis

The chromosomal location information from *G. hirsutum* was visualized by Tbtools software according to the positional information provided in the genome annotation document [31]. The Motif-based sequence analysis tools (MEME, http://meme-suite.org/, accessed on 25 Janyary 2022) were used to analyze the conserved motifs [37]. Then, the phylogenetic tree, conserved motifs, and gene structure were visualized using Tbtools software with the MAST file from the MEME website, the Newick file from TBtools, and the gff3 genome file of *G. hirsutum* [31]. For genes that did not contain UTR regions, we used CottonFGD (https://cottonfgd.org/analyze/, accessed on 25 January 2022) for further verification. The sequence in the Select Analysis option was selected and the 5′ UTR sequence and 3′ UTR sequence that appeared below were checked [33].

### 2.5. Transcriptome Data-Based Gene Expression Analyses of the CesA Gene Family in G. hirsutum L. and G. barbadense L. Tissues

Transcriptome data (https://www.ncbi.nlm.nih.gov/bioproject/PRJNA490626/, accessed on 25 January 2022) for *G. barbadense* Hai7124 and *G. hirsutum* TM-1 tissues, including 0 days post-anthesis (0 DPA), 1 DPA, 3 DPA, and 5 DPA, fiber at 10 DPA, 20 DPA, and 25 DPA, were downloaded from a previous study [38]. Log_2_(FPKM+1) normalization was performed on the expression data. Finally, the standardized data were drawn with Tbtools software [31].

### 2.6. DNA Extraction, RNA Isolation, and qRT-PCR

In the molecular identification of *Arabidopsis*-positive seedlings, the genomic DNA of *Arabidopsis* was extracted using the cetyl-trimethylammonium bromide (CTAB) method [39]. We collected the ovules at 0 DPA and 5 DPA, and fibers at 10 DPA, 20 DPA, 25 DPA, and 30 DPA from *G. hirsutum* TM-1 and the ovules at 0 DPA and fibers at 10 DPA, 20 DPA, 25 DPA, and 30 DPA from *G. barbadense* Hai7124. All samples were frozen in liquid nitrogen and stored at −80 °C. Total RNA was extracted by the RNAprep Pure Plant Plus Kit (Polysaccharides&Polyphenolics-rich) (DP441) (Tiangen, Beijing, China). Then, a reverse transcription kit StarScript Ⅱ First-strand cDNA Synthesis Mix With gDNA Remover StarScript Ⅱ cDNA (GenStar, Beijing, China) was used for cDNA synthesis. The fluorescent quantitative kit was the 2×RealStar Green Fast Mixture with ROX Ⅱ (GenStar, Beijing, China). The experimental design included 3 biological and 3 technical replicates for each gene, and the relative gene expression data were calculated by the 2^−ΔΔCT^ method [40]. The candidate gene sequence, a relatively specific primer for real-time fluorescence quantitative PCR, was designed by NCBI primer-blast, and the amplification product of GhMCesA35 was 247 bp, respectively. The *G. hirsutum* L. His3 (GhHis3) and *A. thaliana* actin (AtActin) genes were used as the reference control, the primer sequences of which are listed in Table S1.

### 2.7. Transformation of A. thaliana

For further analysis, the function of the GhMCesA35 (GhM_D10G0367) gene, the full-length cDNA of GhMCesA35, was amplified from 0 DPA fiber cDNA of *G. hirsutum* with the specific primers. Then, the amplified product was inserted into a PRI101 vector digested with *Sal* *Ⅰ* and *BamH* *Ⅰ* using the one-step Cloning Kit (Vmazyme, Nanjing, China, C112-01). The 35S: GhMCesA35 plasmid was introduced into Col-0 *Arabidopsis thaliana* using Agrobacterium GV3101, and transformants were selected on a medium containing kanamycin (50 mg/L) [41]. All of the primers used in this study are listed in the Supplementary Materials Table S2.

### 2.8. Detection of Cellulose Content in Arabidopsis thaliana

In the *Arabidopsis* T3 generation, 3 healthy plants were selected from 3 positive lines after 6 weeks of growth in the soil. To minimize the experimental error, the selected wild-type *Arabidopsis* and the positive seedlings were grown in the same incubator and tray. After molecular identification, we chose the aboveground part of the plant’s main stem, removed the fruit pods and flowers, and quickly froze the samples in liquid nitrogen. Then, the samples were delivered to the company (RuiYuan, Nanjing, China) for cellulose content determination with the plant cellulose ELISA kit. Since the sampling already contained three biological replicates, each treatment only contained three technical replicates in the determination.

### 2.9. Yeast Double Hybrid and Point-To-Point Verification, and Interaction Network Prediction

Taking the amino acid sequence of GhMCesA35 as the query sequence, the online website STRING was used to predict the interaction, and Cytoscape was used to arrange the prediction results [42]. We conducted point-to-point verification on one of the genes named GhCesA7 (Ghir_D07G004340), constructed the pGBKT7 vector, and constructed the pGADT7 vector for GhMCesA35. The pGBKT7 vector was used to study the transcriptional activity of the CesA7 (Ghir_D07G004340) protein in yeast. The CDS sequence of CesA7 (3129 bp) was amplified from 0 DPA fiber cDNA of *G. hirsutum* with the specific primers (Table S1). Then, the CDS sequence was subcloned to structure the recombinant plasmids, named pGBKT7-CesA7. Then, the recombinant plasmids, pGBKT7-P53 + pGADT7-T and pGBKT7-lam + pGADT7-T, were used as positive and negative controls; other control vector plasmids, including pGADT7 + GhMCesA35-pGBKT7, pGADT7 + pGBKT7-CesA7, and pGADT7 + pGBKT7, were transformed into the yeast strain Y2H-Gold. The transformants were cultured in a synthetically defined medium SD/-Trp/-Leu (DDO) and incubated in a 30 °C electrothermal constant temperature incubator for 3–5 days. Subsequently, the 4 transformants were transferred to a synthetically defined medium SD/-Trp/-His/-Ade/-Leu (QDO) supplemented with X-α-GAL/ABA and incubated at 30 °C for 3–5 days.

### 2.10. Cold, Drought, and Salt Stress Treatment

TM-1 seedlings were grown in a greenhouse at 25 °C with a 16-h light/8-h dark cycle until the 3-leaf stage for cold treatment. Then, the plants were grown in an incubator at 4 °C with a 16-h light/8-h dark cycle. As for the drought and salt treatment, we chose seeds that had been shed evenly and placed them on moist filter paper for germination until two cotyledons emerged. Then, vigorous and uniform cotton seedlings were selected and transferred to 1/2 Hoagland nutrient solution [43] for greenhouse hydroponic culture under conditions consisting of a 16-h light/8-h dark cycle at 25 °C. The nutrient solution was changed every three days to ensure healthy seedling growth. Once the cotton seedlings reached the 3-leaf stage, the different seedlings were treated with 15% PEG6000 and 150 mM NaCl [44]. Finally, samples were harvested from the same leaf at 5 time points (1, 3, 6, 12, 24 h) and immediately frozen in liquid nitrogen, and the control was the untreated plants.

## 3. Results

### 3.1. Dentification and Sequence Analysis of the Cotton Cellulose Synthase Gene Family

In this study, to comprehensively identify the CesA protein in tetraploid cotton species, we analyzed the newly sequenced tetraploid cotton specie AD1 (*G. hirsutum*).

After a series of analyses, we identified 38 sequences in *G. hirsutum* (NDM8). To understand the CesA gene family more conveniently, the CesA genes were renamed according to the gene’s position on the cotton chromosome. For example, we assigned the name GhMCesA1-GhMCesA38 to CesA genes for *G. hirsutum* (Supplementary Materials Table S1). We also analyzed the biochemical properties of CesA proteins, including the gene lengths, isoelectric point (pI) values, and molecular weights (MWs) (Supplementary Materials Table S1). For *G. hirsutum*, the isoelectric point (pI) varied from 5.1 (GhMCesA6) to 9 (GhMCesA32), with an average of 6.88, and MWs varied from 15.43 (kDa) to 123.59 (kDa), with a mean of 101.42 (kDa). GhMCesA35 was located in the region 3553505–3558276 of the D10 chromosome. The CDS sequence length of GhMCesA35 was 2925 bp.

### 3.2. Phylogenetic Analysis and Chromosomal Distribution of CesA Genes

**Figure 1:**
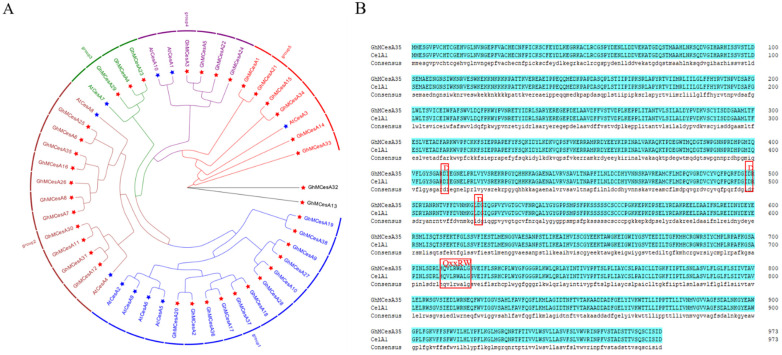
Phylogenetic analysis of CesA genes. (**A**) Phylogenetic analysis of CesA genes in *G. hirsutum* L. and *A. thaliana*. The phylogenetic tree was constructed by TBtools software with the Maximum Likelihood (ML) method with 1000 bootstrap replicates. The CesA genes from *G. hirsutum*, Arabidopsis, are marked with a red, blue five-pointed star. (**B**) Multiple sequence alignment of domains containing the D, D, D, QxxRW motifs in *G. hirsutum* L. (GhMCesA35 and CelA1).

To evaluate the evolutionary relationship of CesA proteins among *G. hirsutum* and *A. thaliana*, phylogenetic analysis of 48 protein sequences was performed to construct the phylogenetic tree based on multiple sequence alignment using the maximum likelihood (ML) method in tbTBtools with 1000 bootstrap replicates, including 38 in *G. hirsutum* and 10 in *A. thaliana* (Figure 1A). After that, we divided those CesA genes into 5 groups (expect GhMCesA32 and GhMCesA13), where the first group had 16 genes, the second group had 13 genes, the third group had 4 genes, the fourth group had 6 genes, and the fifth group had 7 genes. GhMCesA32 and GhMCesA13 were not grouped because of their low homology with other genes. However, we found through BLAST that these two genes had the highest homology with AtCesA3 (AT5G05170). The GhMCesA35 in *G. hirsutum* and its homologous AtCesA8 in *A. thaliana* were well clustered in group 2. CelA1 has previously demonstrated that binding UDP-G can participate in cellulose synthesis in vitro and is specifically expressed during cotton fiber development. Therefore, we matched the protein sequences of GhMCesA35 and CelA1, and the result showed that two sequences were completely consistent, with D, D, D, and QxxRW motifs (Figure 1B).

**Figure 2:**
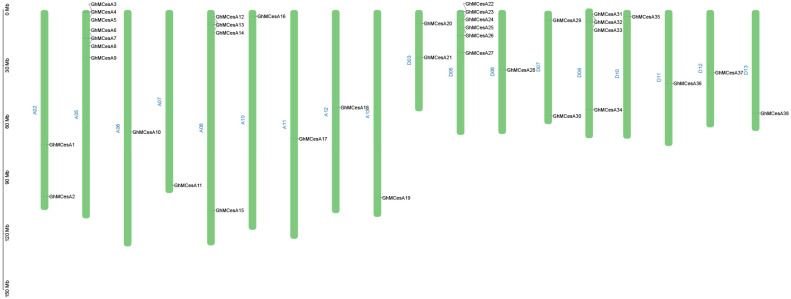
Chromosomal distribution of CesA genes in *G. hirsutum* L. The scale represents mega-bases (Mb).

The mapping of 38 CesA genes to chromosomes based on the available genomic information on *G. hirsutum* revealed that the CesA genes were evenly distributed on the chromosomes. The results are shown in Figure 2. In Gossypium, 38 CesAs were located in 18 chromosomes (Figure 2). The mapped chromosomes were A02/A05/A06/A07/A08/A10/A11/A12/A13 and D03/D05/D06/D07/D08/D10/D11/D12/D13. The number of genes on the mapping chromosomes ranged from 1 to 7. However, GhMCesA35 (GhM_D10G0367) was located on chromosome 10 of the D subgenome, and this chromosome contains only 1 CesA gene.

### 3.3. Gene Structure and Protein Domains Analysis of CesA Genes

**Figure 3:**
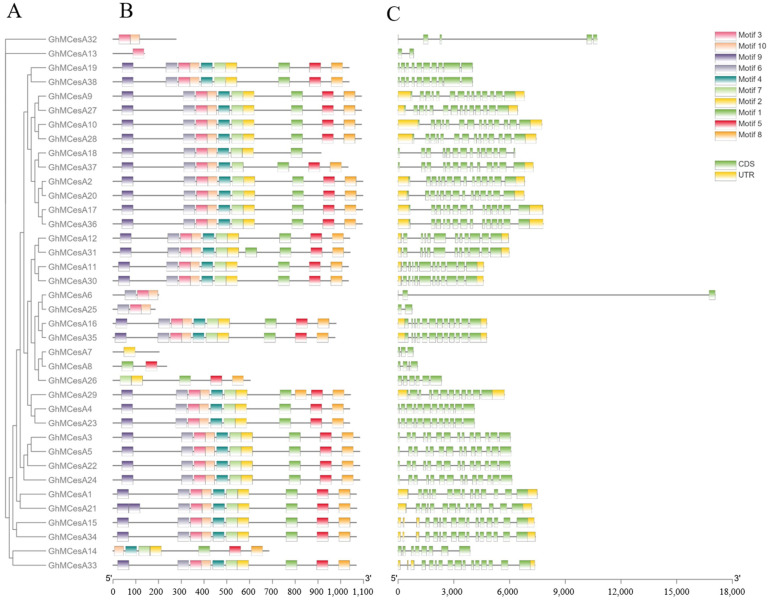
Phylogenetic tree, motif, and gene structure of GhMCesA genes in *G. hirsutum*. (**A**) Phylogenetic analysis of the amino acid sequence of CesA in *G. hirsutum*. (**B**) Conserved motifs of CesA proteins in *G. hirsutum*. (**C**) The gene structures of the CesA genes. The yellow rectangle represents CDS. The green rectangle represents UTR.

The diversity of the gene structure and the differentiation of conserved motifs are possible mechanisms for the evolution of polygenetic families [45]. Interestingly, 17 genes had no UTR, including GhMCesA32, GhMCesA13, GhMCesA19, and GhMCesA38 (Figure 3C). Since we identified this gene family based on the genome sequence of HEBAU_NDM8, Figure 2 only shows the results in the genome of HEBAU_NDM8. However, we examined the UTR region in CottonFGD for these 17 genes in different *G. hirsutum* genome assembles (Table S3). We identified the conserved motifs of CesA proteins and idneitifed 10 conserved motifs (named motif 1 to motif 10). The results are represented in schematic diagrams (Figure 3B). According to the results, some genes have similar motifs, indicating that the same family genes had similar functions. A motif is a structural component with a specific spatial conformation and function in a protein molecule. It is a subunit of a structural domain and is associated with a specific function. Some gene structures of the CesA genes family might have changed during evolution and may have a more important function in cotton growth and development than originally thought.

### 3.4. Tissues-Specific Expression and qRT-PCR Analysis of CesA Genes in G. hirsutum L. and G. barbadense L.

**Figure 4:**
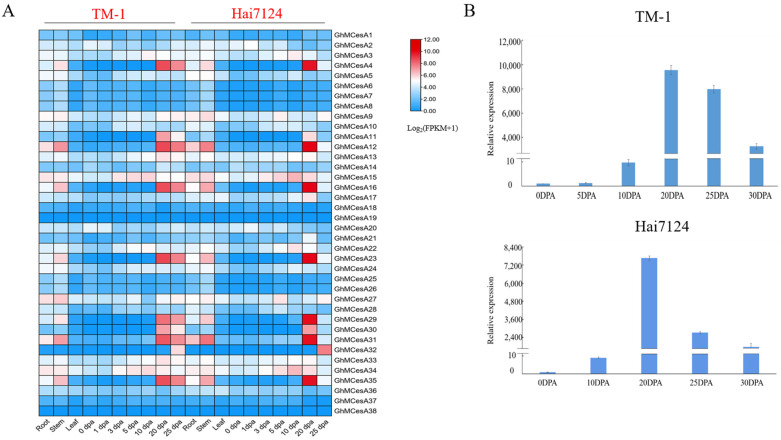
Tissue-specific expression profiles of CesA genes in different tissues of TM-1 and Hai7124. (**A**) Transcriptome data expression heat map. The data was processed with Log_2_(FPKM+1). (**B**) Relative expression of GhMCesA35 in TM-1 and Hai7124.

Due to the better fiber quality in *G. barbadense*, to better reveal the tissue-specific expression profiles of CesA genes in the development process of cotton fiber, fiber expression data of the different development stages in *G. hirsutum* and *G. barbadense* from a public database were used for the analysis. Then, a heat map was constructed with these transcriptome data (Figure 4A). As we can see, the 38 cellulose synthase genes displayed different expression patterns. However, the same gene in another cotton generally showed a similar expression pattern.

GhMCesA35 showed higher expression during fiber secondary wall biosynthesis development in TM-1 and Hai7124. So, we further verified the expression pattern of GhMCesA35 in the above during fiber development in *G. hirsutum* and *G. barbadense*, using the fiber or ovule cDNA as templates for real-time fluorescence quantitative PCR (qRT-PCR). After the qRT-PCR verification of GhMCesA35 in the cDNA of TM-1 and Hai7124, respectively, we found that the expression pattern of GhMCesA35 in TM-1 and Hai7124 was the same, and both were highly expressed during the fiber secondary wall biosynthesis period (Figure 4B). Therefore, GhMCesA35 may play an essential role in the later stage of cotton fiber development.

### 3.5. Determination of Cellulose Content in Stems of Arabidopsis

**Figure 5:**
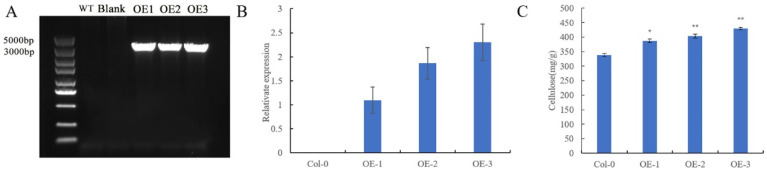
Identification of GhMCesA35 transgenic plants by PCR. (**A**) The PCR confirmation of GhMCesA35 in three transgenic lines of *Arabidopsis*. The primer used was 35S:PRI101-F and PRI101-GhMCesA35-R (Supplementary Materials Table S2). The “N” represents the use of sterile water as a PCR template. (**B**) The relative expression level of GhMCesA35 in three transgenic *Arabidopsis* lines. The ΔCt value of GhMCesA35 in transgenic line 1 was set as the control. The data presented are the means ± SD of three biological replicates. (**C**) The cellulose content of GhMCesA35 in three transgenic lines. The significance of difference was analyzed with the two-tailed t test (* 0.01 < *p* < 0.05, ** *p* < 0.01). Data are represented as average values with SD.

Since the cellulose synthase genes directly act on cellulose synthesis, the functional study of GhMCesA35 started with the cellulose content of transgenic *A. thaliana*. Through the screening of *Arabidopsis* transgenic plants, 3 T3 transgenic-positive plants were obtained, numbered OE-1, OE-2, and OE-3, respectively. Using wild-type *A. thaliana* stalks, and 3 transgenic *Arabidopsis* lines OE1, OE2, and OE3 as materials, DNA and RNA extraction and cDNA synthesis were carried out. To identify *Arabidopsis*-positive seedlings more accurately, we used 35S:PRI101-F and PRI101-GhMCesA35-R (*BamH Ⅰ*) to identify *A. thaliana* DNA (Supplementary Materials Table S2). The result of the DNA amplification is shown in Figure 5A. The length of the target band is consistent with our expected band length of GhMCesA35. To overcome the potential problem of measuring the cellulose content, it is vital to ensure that comparisons are made at the same developmental stage. Then, we used AtActic-F and AtActin-R as internal reference primers to quantitatively verify OE-1, OE-2, and OE-3 and found that they are indeed positive transgenic strains and usually express GhMCesA35 (Figure 5B). Next, the stalks of *A. thaliana* grown in the soil for six weeks (with the fruit pods and inflorescences removed) were used to determine the cellulose content. The above results indicate that GhMCesA35 was successfully transformed into *A. thaliana* and is usually expressed. The cellulose content results showed that the cellulose content of the transgenic line was significantly higher than that of the wild type, and it was consistent with the expression level of GhMCesA35. This result indicates that GhMCesA35 can promote cellulose synthesis, as shown in Figure 5C. Combined with its expression in the fiber, it is speculated that GhMCesA35 plays a vital role in developing cotton fiber.

### 3.6. GhMCesA35 Interacted with GhCesA7

**Figure 6:**
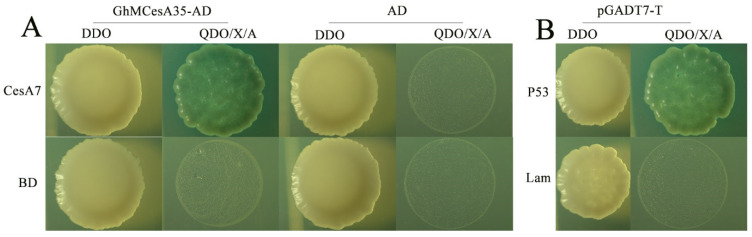
Yeast two-hybrid assay of GhMCesA35–GhCesA7 interaction. (**A**) Physical interaction of GhMCesA35 with CesA7 in yeast cells. CesA7: Ghir_D07G004340. (**B**) pGBKT7-P53+pGADT7-T and pGBKT7-lam+pGADT7-T were used as positive and negative controls. The yeast co-transformed the positive control vector grown on SD-Trp-Leu-His-Ade/X-α-Gal/ABA(QDO/X/A) medium. CesA7-BD: GhCesA7-pGBKT7, GhMCesA35-AD: GhMCesA35-pGADT7, BD: pGBKT7, AD: pGADT7, BD: pGBKT7, P53: pGBKT7-P53, lam:pGBKT7-lam, DDO: SD-Trp-Leu, QDO/X/A: SD-Trp-Leu-His-Ade/X-α-Gal/ABA.

By predicting the results, we constructed an interaction network and used GhCesA7 as the candidate gene (Supplementary Materials Figure S1). Moreover, our results showed that the experimental group (GhMCesA35-AD + CesA7-AD) and the positive control (pGBKT7-p53 + pGADT7-T) grew well on SD-Trp-Leu (DDO) and SD-Trp-Leu-His-Ade/X-α-Gal/ABA (QDO/X/ABA) medium (Figure 6A). However, the negative controls grew on the DDO medium but could not grow on the QDO/X medium (Figure 6B). The above results showed that GhMCesA35 interacted with GhCesA7.

### 3.7. qRT-PCR Analysis of GhMCesA35 under Different Stress Treatments

**Figure 7:**
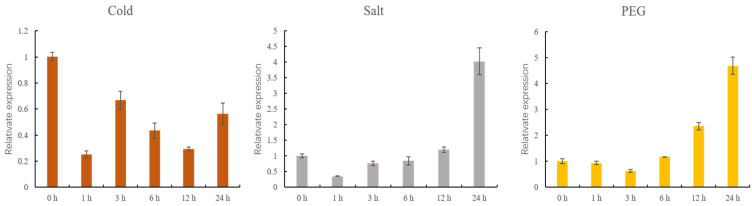
qRT-PCR analysis of GhMCesA35 under cold, salt, and PEG stress treatment. Data are represented as average values with SD. The ΔCt value of GhMCesA35 in transgenic line 1 was set as the control.

To examine the expression of GhMCesA35 under different stress treatments, we set up 3 stress treatments, and samples were taken within 0, 1, 3, 6, 12, and 24 h. After cold stress treatment, we found that the expression level of GhMCesA35 was lower than the control treatment (Figure 7). However, under the drought and salt stress treatment, the expression level of GhMCesA35 was higher than that of the control treatment (Figure 7).

## 4. Discussion

Upland cotton is the most widely planted fiber crop globally due to its high fiber yield. However, the fiber quality of upland cotton is not as good as *Gossypium barbadense*. Therefore, improving the fiber quality in upland cotton is essential for cotton growers. Fiber is made up of cellulose, which is the main component of the cell wall. Because of the vital role of the cell wall in fiber development, investigation of cellulose synthesis is essential for the improvement of fiber quality. So, understanding the functions of cellulose synthase in upland cotton is of great value.

Five allopolyploid cotton species have been sequenced and assembled in recent years, including economically significant upland cotton [30,46]. Therefore, we used the genome of upland cotton from previous studies and identified 38 CesA genes in upland cotton. After checking the gene structure of these 38 CesA members, we noticed that the exons, introns, and motifs of these members were similar, further illustrating the validity of the CesA identification. This result filled in the blank of the CesA gene in *G. hirsutum* acc NDM8 (AD1). The protein sequence of GhMCesA35 is precisely the same as that of CelA1 previously characterized (Figure 1B).

Only expressed genes have the potential to be transcribed and translated into proteins, so we performed expression profiling of CesA members during fiber development in TM-1 and Hai7124. The expression profiling results showed that GhMCesA35 had higher expression at 20 DPA and was furtherly validated by qRT-PCR (Figure 4A,B). These results indicate the potential role of GhMCesA35 in secondary cell wall synthesis.

In previous research findings, we found that CelA1 is involved in secondary cell wall biosynthesis and binds UDP-glucose in vitro [27]. However, the function of CelA1 had not been validated in vivo. So, the overexpressing *Arabidopsis thaliana* in this study demonstrated the function of GhMCesA35 in cellulose synthesis. On the other hand, previous studies proposed that cellulosic synthase complex (CSC) contains 18, 24, or 36 CesAs [17,47]. In the CSC, the loss of cellulosic synthase members will affect the assembly of CSC, and thus affect the synthesis of cellulose [48]. A previous article reported that CesA4, CesA7, and CesA8 of *Arabidopsis thaliana* are co-expressed in the same cells, and all three proteins interact in detergent-solubilized extracts, which suggests CesA4, CesA7, and CesA8 can form a heterotrimer [49]. The wet-lab experiment also indicated that GhMCesA35 interacted with GhCesA7. As a member of cellulose synthase, we inferred that GhMCesA35 may also interact with other CesA members to form a complex in fiber cells.

The expression pattern of the treated GhMCesA35 and CK groups indicates the involvement of GhMCesA35 in salt and drought tolerance. In previous studies, cellulose in the cell wall was essential for plant morphogenesis and response to specific external stimuli [11,26,50,51]. In response to abiotic stress, some plants adopt a strategy of increasing the thickness of secondary cell walls through the synthesis of cellulose, which helps to improve the hydration status of the plant and maintain the turgor pressure required for growth [52,53]. Finally, we inferred that GhMCesA35 could enhance the strength of the cell wall, which holds the cell shape and may tolerate some abiotic stresses, such as salt and drought.

## 5. Conclusions

In summary, we verified that GhMCesA35 is involved in cellulose biosynthesis, and also identified interaction with GhCesA7, laying the foundation for further investigation of the composition of CSC in the future. Our research work provides new ideas and methods for studying the biological functions of CesA genes in *G. hirsutum*, thereby improving cotton fiber quality and yield.

## Data Availability

The data presented in this study are available in the article and Supplementary Materials.

## Supplementary Materials

The following supporting information can be downloaded at: https://www.mdpi.com/article/10.3390/genes13020292/s1, Supplement Figure S1A: The Interaction network between GhMCesA35 and different genes. Supplement Figure S1B: Gene IDs that may interact with GhMCesA35 were predicted. Supplement Table S1: The gene features of 38 CesA genes in *G. hirsutum*. Supplement Table S2: Primers used in this study. Supplement Table S3: Identification of UTR regions in 17 GhMCesA genes in five genomes. Supplement Table S4: Gene IDs and numbers involved in each step during the identification process.
