# Supplementary figures and images for: Identification of the CesA Subfamily and Functional Analysis of GhMCesA35 in Gossypium hirsutum L
Source: Genes (Basel). 2022 Feb 1;13(2):292. doi: 10.3390/genes13020292 (PMC8871739; doi:10.3390/genes13020292)

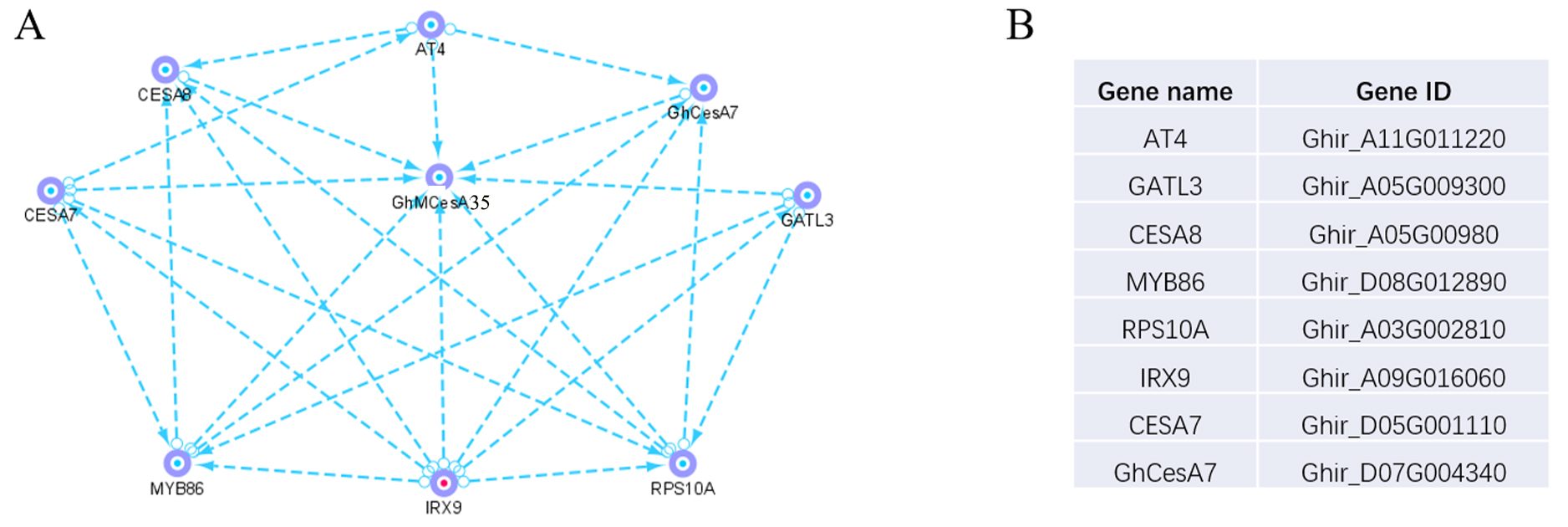

Supplement: Supplementary file 1 [file genes-13-00292-s001.zip › Figure S1.jpg]
